# Myostatin and markers of bone metabolism in dermatomyositis

**DOI:** 10.1186/s12891-021-04030-0

**Published:** 2021-02-05

**Authors:** Katharina Kerschan-Schindl, Wolfgang Gruther, Ursula Föger-Samwald, Christine Bangert, Stefan Kudlacek, Peter Pietschmann

**Affiliations:** 1 Department of Physical Medicine and Rehabilitation and Occupational, Medical University of Vienna, Waehringer Guertel 18-20, 1090 Vienna, Austria; 2 healthPi - Medical Center, Vienna, Austria; 3 Department of Pathophysiology and Allergy Research, Center of Pathophysiology, Infectiology and Immunology, Medical University of Vienna, Vienna, Austria; 4 Department of Dermatology, Medical University of Vienna, Vienna, Austria; 5 Medizinische Abteilung, Krankenhaus Barmherzige Brüder, Vienna, Austria

**Keywords:** Dermatomyositis, Myostatin, FGF23, Muscle strength, Physical function

## Abstract

**Background:**

In dermatomyostis (DM) patients, inflammation, reduced activity, and medication have a negative impact on the musculoskeletal system. Several endocrine factors are involved in muscle growth and bone turnover.

Objective: We aimed to investigate factors regulating myogenesis and bone metabolism and to evaluate possible associations between these endocrine factors, muscle strength, and functional tests in DM patients.

**Methods:**

We conducted a cross-sectional study in 20 dermatomyositis patients. Serum levels of myostatin (MSTN), follistatin (FSTN), dickkopf 1 (Dkk1), sclerostin (SOST), periostin (PSTN), the receptor activator nuclear factor kB ligand (RANKL):osteoprotegerin (OPG) ratio and fibroblast growth factor 23 (FGF23) were determined. Physical function was evaluated by hand-held strength measurement, chair rising test, timed up and go test and the 3-min walking test.

**Results:**

Serum MSTN and FGF23 levels (2.5 [1.9; 3.2] vs. 1.9 [1.6; 2.3] and 2.17 [1.45; 3.26] vs. 1.28 [0.79; 1.96], respectively; *p* <  0.05) were significantly higher in DM patients than in controls. Dkk1 was significantly lower (11.4 [6.9; 20.0] vs. 31.8 [14.3; 50.6], *p* <  0.01). Muscle strength and physical function tests correlated with each other (e.g. hip flexion – timed up and go test: *r* = − 0.748, *p* < 0.01).

**Conclusion:**

In DM patients, biochemical musculo-skeletal markers are altered and physical function shows deficits. All these tests reflect independent of each other different deficits in long-term DM patients which is important for the assessment of DM patients as well as planning of therapeutic interventions in clinical routine.

## Background

Dermatomyositis (DM) is an idiopathic inflammatory myopathy characterized by chronic skeletal muscle inflammation and weakness; proximal muscles are the ones which are mostly affected. The skin manifestation is expressed as rashes. The aetiology of muscle weakness is not fully understood but besides inflammation, corticosteroid treatment, and disuse, a switch of muscle fibre type as well as decreases of adenosine monophosphate deaminase (AMPD1) may contribute to skeletal muscle weakness in inflammatory myopathy [1, 2]. Inflammation, corticosteroid treatment, and low body weight may also be factors responsible for the elevated susceptibility to osteoporosis and fragility fractures in DM patients [3, 4].

Throughout life muscle metabolism is influenced by several endocrine factors. One of the key factors regulating myogenesis is myostatin (MSTN). Much interest has been given to this negative regulator of muscle mass and its inhibitor follistatin (FSTN) in physiologic as well as pathophysiologic conditions, including muscular dystrophies and inflammatory myopathies [for review see [5]]. Just like muscle metabolism, bone homeostasis also depends on the balance between positive and negative regulators. Osteocytes play a central role in the regulation by secreting different proteins: Sclerostin (SOST) and dickkopf-1 (Dkk 1), inhibitors of the Wnt/ß-catenin signaling pathway, which reduce bone formation and regeneration, as well as fibroblast growth factor 23 (FGF23), an important regulator of phosphate and vitamin D, thus, being involved in skeletal metabolism [for review see [6]]. Another regulator of bone metabolism is the osteoblast-specific factor 2, periostin (PSTN) which is supposed to be associated with bone strength [7]. The RANKL (receptor activator of nuclear factor-kB ligand) /OPG (osteoprotegerin) system plays an important role in osteoclastogenesis as well [8].

Pathophysiological mechanisms of DM-associated muscle and bone strength loss are not well investigated in DM patients. Only one study [9] evaluated the above-mentioned regulators of muscle growth in DM patients so far. Thus, the aim of this study was to investigate the pathophysiological role of important factors regulating myogenesis and bone metabolism in patients with DM. The primary hypothesis was an inflammation-induced increase of serum levels of myostatin. Additionally, we wanted to evaluate possible associations between these regulators and physical function to increase the understanding of myokines and osteokines involved in the development of musculoskeletal deficits of DM patients.

## Methods

### Study population

Patients with dermatomyositis according to the Bohan and Peter criteria [10, 11] and regular follow-up at the Department of Dermatology, Medical University of Vienna were invited to participate in this cross-sectional study if the diagnosis was verified by muscle biopsy and if the patients were at least 18 years of age. To be eligible, participants had to be able to follow the study protocol. Excluded were patients who had a malignant disease within the previous 5 years, had to undergo surgery within the past 3 months, were immobilized, or had renal or liver insufficiency. The study protocol was approved by the Medical University of Vienna Ethics Committee. The procedure was explained to all participants and written informed consent was obtained.

### Procedures

Anthropometric measures of patients with DM either on standard therapy (glucocorticoids and methotrexate) or intravenous immunoglobulin (IVIG) treatment were performed. Standing height was measured in stocking feet to the nearest centimeter using a stadiometer, and weight was measured using a balance beam scale, recalibrated monthly. Afterwards venous blood samples were drawn. To eliminate diurnal variations in biochemical variables sample collection was performed in the morning. Blood samples were frozen and stored at − 70 degrees after centrifugation until assayed in a single batch run. Additionally, muscle strength was measured and patients conducted three different functional tests.

### Biochemistry

Serum levels of C-reactive protein (CRP), creatinine kinase (CK), and aldolase were measured to give information on disease activity. Basic serum chemistry included calcium, phosphate, creatinine, parathyroid hormone, and 25-hydroxyvitamin D (25OHD). Evaluated bone formation markers were bone specific alkaline phosphatase (BAP) analysed with the Liaison Analyzer (DiaSorin Inc., USA with a detection limit of 0.1 μg/L; an intra-assay coefficient of variation between 3.3 and 4.3%, and an inter-assay coefficient of variation between 6.1 and 8.1%), osteocalcin (Oc) analysed with the Cobas 8000 Analyzer (Roche Diagnostics, Switzerland with a detection limit of 0.01 ng/mL; an intra-assay coefficient of variation between 0.9 and 1.3%, an inter-assay coefficient of variation between 1.2 and 2.3%), and N-terminal propeptide of type I collagen (P1NP) using the Cobas 8000 Roche Analyzer (Roche Diagnostics, Switzerland with a detection limit of 5 ng/mL; an intra-assay coefficient of variation between 1.6 and 3.5%; an inter-assay coefficient of variation between 2.0 and 3.8%). The bone resorption marker cross-linked-C-telopeptide of type I collagen (CTX) was determined with the Cobas 8000 Roche Analyzer (Roche Diagnostics, Switzerland with a detection limit of 0.5 ng/mL; an intra-assay coefficient of variation between 1.2 and 4.7%, an inter-assay coefficient of variation between 1.5 and 5.7%). We performed all biochemical analyses according to standard procedures.

We additionally evaluated some musculoskeletal markers: *myostatin* (MSTN) using a colorimetric competitive immunoassay (Immundiagnostik, Bensheim, Germany with an intra-assay coefficient of variation below 12%, an inter-assay coefficient of variation below 14%; limit of blank LoB: 0.370 ng/ml, according to manufacturer’s data), *follistatin* (FSTN) with a colorimetric sandwich immunoassay (R&D Systems, Minneapolis, USA with an intra-assay coefficient of variation below 3%, an inter-assay coefficient of variation below 10%; MDD range 0.005–0.068 ng/mL; mean MDD 0.016 ng/mL, according to manufacturer’s data), *sclerostin* (SOST, BI-20492) and dickkopf 1 (Dkk 1; BI-20412) using colorimetric sandwich immunoassays by Biomedica, Vienna, Austria (SOST: intra-assay coefficient of variation: ≤7%, inter-assay coefficient of variation: ≤10%, detection limit: 3.2 pmol/l; Dkk 1: intra-assay coefficient of variation: ≤8.0%, inter-assay coefficient of variation: ≤12.0%, detection limit: 0.38 pmol/l, according to manufacturer’s data), and *periostin* (PSTN; SEH339Hu) with colorimetric sandwich immunoassays (Cloud-Clone-Corp, Houston, USA with an intra-assay coefficient of variation ≤10% and an inter-assay coefficient of variation ≤12%;; detection limit: 0.068 ng/ml, according to manufacturer’s data). *Receptor activator nuclear factor kB ligand* (RANKL) and *osteoprotegerin* (OPG) were measured using a commercially available immunoassay from Biomedica, Austria (OPG detection limit: 0.14 pmol/l; intra-assay CV: 4–10%, inter-assay coefficient of variation: 7–8%; RANKL detection limit: 0.08 pmol/l; intra-assay CV: 3–5%, inter-assay coefficient of variation: 6–9%). Serum levels of *fibroblast growth factor 23* (FGF23) were determined by an ELISA targeting the C-terminal end of the molecule (Biomedica, Vienna, Austria). The detection limit of the assay is 0.08 pmol/L with an intra-assay coefficient of variation of ≤12%.

The biomarkers were also assessed in a healthy age matched control group without any medication affecting muscle or bone metabolism. All subjects of the control group were part of a previously published population-based cohort [12].

### Muscle strength

Hand-held muscle strength measurement has been shown to be a feasible and reliable method for evaluation of muscle strength in dermatomyositis patients with strong intra- and interobserver correlations for all muscle groups [13]. Thus, maximal isometric muscle strength of shoulder abduction (SAb), elbow flexion (EF), elbow extension (EE), hip flexion (HF), and knee extension (KE) was evaluated with the handheld dynamometer Hoggan microFET2® (Hoggan Scientific, LLC, Salt Lake City UT, USA). Stabilization specifics used for each muscle action were similar to those performed by Andrews and coauthors [14]. Within 2 s the patient tried to reach maximal isometric strength and held that level for another 4–5 s. The mean value of the left and right sided maximal strength was used for statistical analysis.

### Functional assessment

Three functional tests were performed as part of the physical examination of our study participants. The chair rising test (CRT) was used to assess leg power; it measures the time an individual needs to stand up and sit down five times as quickly as possible from a chair of standard height (46 cm seat height) with his/ her arms fold across the chest [15]. Mobility performance was evaluated by the timed up and go test (TUG), a test which measures, in seconds, the time it takes the individual to stand up from a chair, walk a distance of 3 m, turn around, walk back to the chair, and sit down again [16]. Completion of both tasks was timed from the beginning of the manoeuvre until the patient was re-seated. To assess functional exercise capacity the distance covered within 3 min of walking at self-selected speed corresponding a perceived exertion rate of 13 (“somewhat hard”) on a level course was collected (3-min walking test; 3MWT) [17].

### Statistical analysis

All data are given as medians [quartiles]. Statistical differences concerning BMI and the serum levels of the biochemical markers were assessed with the Mann Whitney U test. Spearman’s rank correlation coefficient was calculated between FGF23 and biochemical markers, between MSTN and muscle strength as well as physical function, and between maximal muscle strength and physical function. A *p*-value less than 0.05 was considered significant. The software packages GraphPad Prism 5 (Prism 5 für Windows, Version 5.00, 2007) and SPSS Statistics V21 (SPSS Inc., Chicago, IL, USA, 2012) were used for statistical analyses.

## Results

Eighteen female and two male DM patients with a median age of 66 [55; 76] years participated in this study. Their disease duration was 6.5 [4.25; 12.5] years. Eight patients were on the traditional medical regimen of glucocorticoid therapy (12.5 [4.7; 15.6] mg/day); half of these patients also received steroid-sparing agents. Twelve patients received intravenous immunoglobulins (IVIG) on a regular basis (2 g/kg for 2–5 days every 4 weeks) but except for two all of these 12 patients additionally needed glucocorticoids and/or immunosuppressive therapy. Three patients of the glucocorticoid group and four patients of the IVIG group took bisphosphonates on a regular basis. Twenty healthy controls – 17 females and three males – with a median age of 61 [56; 68] years were included for comparison of musculoskeletal markers. There was no significant difference between the DM and control group with respect to age, BMI, and bone turnover markers. Serum calcium, phosphate, and creatinine were significantly lower in DM patients compared to the control group. However, all median values were within the normal range. Vitamin D was reduced in both groups; the median value was even lower in the control group.

Serum 25OHD levels were reduced. All other basic chemistry parameters as well as bone turnover markers were within the normal range (Table 1). Maximal isometric muscle strength was lower than published reference values for healthy controls of comparable age. The median time needed to perform the CRT was higher than published reference values. Patients needed 9 s for the TUG test and managed to cover 240 m within the 3 min’ walk (Table 2).

**Table 1:** DM patients’ demographic data and biochemical parameters

	DM patients ***n*** = 20	Control group ***n*** = 20/ Normative values
Age	66 [55; 76]	61 [56; 68]
BMI	26 [23; 30]	26 [23; 30]
Disease duration	6.5 [4.25; 12.5]	–
Treatment		
GC alone	4	–
GC + immunosuppression^c^	4	–
IVIG alone	2	–
IVIG+GC/±immunosuppression^d^	5	–
IVIG+immunosuppression^d^	5	–
CRP [mg/dl]	0.3 [0.1; 0.6]	< 0.5^a^
CK [U/l]	133 [63; 173]	< 170^a^
Aldolase [U/l]	3.8 [2.5; 5.4]	0–7.6^a^
Ca [mmol/l]	2.3 [2.3; 2.4]	2.4 [2.3; 2.6]^b^
Phosphate [mmol/l]	1.04 [0.94; 1.19]	1.20 [1.08; 1.51]^b^
Creatinine [mg/dl]	0.74 [0.64; 0.87]	0.86 [0.79; 1.01]^b^
PTH [pg/ml]	43.2 [33.0; 69.5]	36.0 [15.7; 44.9]
25OHD [nmol/l]	66.7 [50.4; 87.0]	40.7 [26.0; 59.5]^b^
BAP [U/l]	8.8 [5.9; 12.4]	5.2–24.4^a^
OC [ng/ml]	18.8 [10.0; 27.8]	22.3 [15.1; 32.0]
P1NP [ng/ml]	42.0 [29.5; 80.5]	37.7 [28.8; 74.5]
CTX [ng/ml]	0.22 [0.11; 0.50]	0.22 [0.15; 0.33]

*CK* Creatinine kinase, *CRP* C-reactive protein, *PTH* Parathyroid hormone, *25OHD* 25-OH-Vitamin D, *BAP* Bone-specific alkaline phosphatase, *OC* Osteocalcin, *P1NP* N-terminal propeptide of type I collagen, *CTX* Cross-linked-C-telopeptide of type I collagen; median [quartiles]; ^a^: normative values are given; ^b^ *p*<0.05; ^c^: methotrexat (n = 2), azathioprine (*n* = 1), mycophenolatmofetil (n = 1), ^d^: methotrexat (*n* = 5), mycophenolatmofetil (*n* = 2), clycophosphamide (*n* = 1), tacrolimus (*n* = 1)

**Table 2:** DM patients’ physical function

	***n*** = 20	Normative values
Shoulder abduction N [N]	80 [71; 107]	114.1 ± 20.2 [18]^a^
Shoulder abduction D [N]	92 [70; 108]	125.0 ± 25.8 [18]^a^
Elbow flexion N [N]	110 [72; 147]	150.8 ± 26.5 [18]^a^
Elbow flexion D [N]	115 [73; 137]	156.7 ± 29.4 [18]^a^
Elbow extension N [N]	86 [66; 122]	96.6 ± 24.2 [18]^a^
Elbow extension D [N]	81 [62; 113]	96.1 ± 22.9 [18]^a^
Hip flexion N [N]	87 [50; 115]	121.2 ± 21.2 [18^a^]
Hip flexion D [N]	88 [59; 113]	122.7 ± 23.2 [18]^a^
Knee extension N [N]	203 [99; 288]	248.0 ± 66.4 [18]^a^
Knee extension D [N]	207 [77; 281]	257.2 ± 58.0 [18]^a^
CRT [s]	11.8 [7.2; 17.6]	≤ 10 [19]
TUG test [s]	9.0 [5.4; 10.0]	< 10 [20]
3MWT [m]	240 [183; 277]	n.a.

*N* Nondominant, *D* Dominant, *CRT* Chair rising test, *TUG* Timed up and go test, *3MWT* 3 min walking test, *n.a*. Not applicable, median [quartiles]; ^a^ decade and gender-specific data are given as mean ± SD

Group comparison of musculoskeletal markers is shown in Table 3. Serum MSTN und FGF23 levels were significantly higher in DM patients than in controls. DKK 1 was significantly lower in DM patients. Concerning FSTN, SOST, PSTN, and the RANKL:OPG ratio no group-specific differences could be detected.

**Table 3:** Musculoskeletal markers of DM patients and controls

	DM (***n*** = 20)	Controls (***n*** = 20)	***p***-value
MSTN [ng/ml]	2.5 [1.9; 3.2]	1.9 [1.6; 2.3]	< 0.05
FSTN [pg/ml]	1343 [929; 1856]	1232 [1093; 1433]	n.s.
SOST [pmol/l]	28.9 [23.3; 34.4]	28.3 [24.4; 35.7]	n.s.
Dkk 1 [pmol/l]	11.4 [6.9; 20.0]	31.8 [14.3; 50.6]	< 0.01
PSTN [ng/ml]	3.2 [2.0; 5.8]	3.6 [2.6; 7.0]	n.s.
RANKL/OPG	0.017 [0.005; 0.055]	0.030 [0.020; 0.048]	n.s.
FGF23 [pmol/l]	2.17 [1.45; 3.26]	1.28 [0.79; 1.96]	< 0.05

*MSTN* Myostatin, *FSTN* Follistatin, *SOST* Sclerostin. *Dkk 1* Dickkopf 1, *PSTN* Periostin, *RANKL* Receptor activator nuclear factor kB; *OPG* Osteoprotegerin, *FGF23* Fibroblast growth factor 23

Correlation analyses revealed a significant positive association between serum levels of FGF23 and aldolase (*r* = 0.563; *p* < 0.05), phosphate (*r* = 0.572; *p* < 0.05), as well as 25OHD (*r* = 0.553; *p* < 0.05). No association could be detected with creatinine kinase. No statistically significant correlations were found between MSTN on one side and muscle strength and physical function on the other side (r ranged from − 0.426 to 0.194; *p* > 0.05). Correlations between maximal muscle strength obtained by manual muscle testing and functional tests are shown in Table 4.

**Table 4:** Correlations – muscle strength and physical function in DM patients

	SAb	EF	EE	HF	KE	CRT	TUG	3MWT
SAb	1	0.791**	0.464*	0.707**	0.739**	−0.572*	−0.583*	0.529*
EF		1	0.782**	0.648**	0.815**	−0.513	− 0.513*	0.604*
EE			1	0.565**	0.699**	−0.366	− 0.408	0.482*
HF				1	0.788**	−0.729**	−0.748**	0.788**
KE					1	−0.651**	−0.680**	0.633**
CRT						1	0.910**	−0.841**
TUG							1	−0.868**
3MWT								1

*SAb* Shoulder abduction, *EF* Elbow flexion, *EE* Elbow extension, *HF* Hip flexion, *KE* Knee extension, *3MWT* 3 min walking test, *CRT* Chair rising time, *TUG* timed up and go test; * *p* < 0.05; ** *p* < 0.01

## Discussion

This is the first study evaluating myokines and osteokines potentially involved in musculoskeletal disturbances of DM patients. It revealed elevated MSTN and FGF23 serum levels as well as reduced levels of DKK 1.

This investigation’s finding of higher MSTN levels in DM patients compared to controls is in contrast to one study evaluating different myositis subtypes. Vernerova et al. [9] could not reveal significant differences in serum levels of MSTN in their DM patients compared to healthy controls. However, comparison between these two studies is difficult because characteristics of patients are only given for the whole group of idiopathic inflammatory myopathy patients; no separate data of the included DM patients are shown. Potentially important discrepancies between the study populations are the following: Disease duration of our study’s patients was much longer (median: 6.5 years vs. mean: 1.04 years) but inflammation - expressed as CRP and CK - was lower. The negative correlation between MSTN and CRP shown by Vernerova et al. [9] goes in line with the fact that our patients with a lower degree of inflammation had higher serum levels of MSTN.

Considering elevated MSTN levels one would suspect MSTN as a good therapeutic target. However, in several neuromuscular diseases, downregulation of the MSTN pathway has only partially been successful [18–20]. Wagner [21] outlined very well different reasons why MSTN inhibition did not hold what preclinical data promised in muscular dystrophy. One point was concomitant prednisolone treatment. The median glucocorticoid dose of this study’s GC group was 12.5 mg per day and almost half of the IVIG patients additionally needed glucocorticoids. It is known that glucocorticoids stimulate muscles’ production of MSTN [22]. Thus, increased MSTN levels in long-term DM patients may be caused by irreversible muscle damage and/or reduced muscle mass.

This study’s patients had a long disease duration but a low level of inflammation indicated by median values of CRP, CK, and aldolase within the normal range. However, even chronic low-grade inflammation is associated with loss of muscle mass and reduced ability to carry out physical activities. Muscle strength was lower than reference values for healthy subjects. A look at the results of functional assessment gives the impression that physical activity was probably only marginally limited. The association between MSTN and physical function has been investigated in a few studies. Arrieta H et al. [23] evaluating very old and frail people observed lower levels of MSTN in frail women whereas two studies [24, 25] investigating younger and less limited subjects found elevated serum levels of MSTN in physically frail women. In line with the last two studies, this investigation’s DM patients with a median age of 66 years and deficits in muscle strength and physical function had higher MSTN levels than healthy controls. Interestingly FSTN, the antagonist of MSTN was alike to the control group. This finding is in line with the DM subjects investigated by Vernerova et al. [9] and emphasizes the relevance of MSTN.

Up to now, no data on regulators of the Wnt signalling pathway have been investigated in inflammatory myopathies. This study’s results of decreased DKK 1 levels and no group-specific difference of SOST are in accordance with our previous study on RA patients in remission [26]. The reduced and normal levels of the Wnt inhibitors, respectively, foreclose a reduction of bone formation. This assumption is corroborated by the fact that the markers of bone formation were in the normal range. It should also be mentioned that Dkk1 is not as bone specific as SOST because it is expressed in other tissues as well.

Since PSTN levels were like the control group one can assume that these long-term DM patients were well treated and did not have an elevated risk of fragility fractures. This is underlined by normal values of the BTMs and vitamin D. Additionally, some patients took a bone-specific medication.

The RANKL:OPG ratio did not show a significant difference between DM patients and controls. At the time of diagnosis children with juvenile DM had an increased RANKL:OPG ratio [27]. However, another study investigating juvenile DM patients on glucocorticoid therapy with a mean disease duration of 46 months could not detect a difference of the RANKL:OPG ratio compared to healthy controls [28]. Patients participating in this study received IVIG and/ or glucocorticoids - treatments with contrary effects on bone metabolism. Glucocorticoids suppress OPG and increase RANKL expression [29] whereas IVIG is supposed to inhibit osteoclastogenesis by supressing RANKL signalling [30].

We for the first-time evaluated serum FGF-23 in dermatomyositis and related the serum levels to parameters of clinical chemistry and disease activity. Normal values of the clinical chemistry parameters indicated that there was no disturbance in our patients’ phosphate metabolism or renal function. The positive correlation between aldolase and FGF23 indicates that despite the fact of relatively low serum markers of current disease activity, prevalent residual activity of the disease influenced the expression of FGF23. In vitro and experimental studies have shown that pro-inflammatory stimuli increase serum levels of FGF23 [31, 32]. Correlations with inflammatory markers have also been reported in several different inflammatory diseases (for review see [33]) including rheumatoid arthritis - in one of two studies investigating such patients [34, 35]. Therefore, elevated FGF23 levels detected in this study may be caused by DM associated inflammation. Since this study’s patients did not show a highly active disease, patients’ intake of glucocorticoids may also be relevant for the changes in FGF 23 expression. In their investigation Sato and co-authors could not detect a correlation of FGF23 with glucocorticoid doses [34] in rheumatoid arthritis. Nevertheless, an experimental study showed that the release of FGF23 is upregulated by the exposure to deoxycorticosterone acetate [36]. Correlation analyses showed a positive association of FGF23 with serum levels of phosphate, and 25OHD. That fits well with the central role of FGF23 in the regulation of phosphate balance. No data on a potential effect of IVIG therapy on FGF23 exist.

Compared to the mean normative values published by Andrews et al. (1996) [14] median maximal isometric muscle strength in our long-term DM patients was reduced. That is in line with two of three previous studies [37–39] and is most likely caused by the disease associated muscle damage. We have shown that this low muscle strength can be improved by exercise without a negative effect on inflammation [40]. The increased time needed for the CRT certainly is an expression of the reduced leg power caused by muscle damage as well. As our patients managed the TUG test within the normal time limit, they performed better than juvenile DM patients investigated by Berntsen et al. [41]. The median distance covered during the 3MWT was 240 m. Unfortunately, no reference values exist for this test which is related to maximal oxygen uptake [17] but results obtained in this study were better than in a study investigating clinically stable patients with chronic obstructive pulmonary disease (COPD) [42]. A lack of difference in the 6MWT in inactive inflammatory myopathies [43] and inactive juvenile DM [41] has been shown. All muscle strength tests showed a positive moderate to high correlation with each other. As expected, the CRT and the TUG test correlated negatively whereas the 3 MWT correlated positively with the muscle strength measurements. Physical function tests were highly to very highly associated with each other.

There are several limitations to this study. An aspect we could not meet is the evaluation of the different effects of IVIG and glucocorticoids on muscle and bone metabolism. The sample size was too small and pursuing the goal of steroid reduction therapy was heterogeneous. Thus, it was not possible to split the study collective for further statistical analyses. Since our control group was part of a previously published population-based cohort, it was not possible to evaluate muscle strength in the control group. However, muscle strength reference values for healthy controls of comparable age may be even more reliable than data evaluated from a small control group. Biochemical evaluation of inflammation was used instead of a disease-specific activity assessment tool. Additionally, the latest classification criteria [44] were not applied in this study as the patients were recruited before their publication. Anyhow, retrospective analysis revealed that all included patients also fulfilled the EULAR/ACR classification criteria for idiopathic inflammatory myopathies and the classification tree for DM according to Lundberg and co-authors [44].

## Conclusion

We conclude that in patients with long-term dermatomyostis serum levels of myostatin and FGF23 are increased. The study also showed that biochemical musculo-skeletal markers as well as functional tests reflect, independent of each other, different deficits in long-term DM patients.

## Data Availability

All necessary information is contained in the manuscript. The datasets used are available from the corresponding author on reasonable request. Each participant’s raw data are deposited in hospital archive.

## Abbreviations

DM: Dermatomyositis
CK: Creatinine kinase
CRP: C-reactive protein
PTH: Parathyroid hormone
25OHD: 25-OH-Vitamin D
BAP: Bone-specific alkaline phosphatase
OC: Osteocalcin
P1NP: N-terminal propeptide of type I collagen
CTX: Cross-linked-C-telopeptide of type I collagen
MSTN: Myostatin
FSTN: Follistatin
SOST: Sclerostin
Dkk 1: Dickkopf 1
PSTN: Periostin
RANKL: Receptor activator nuclear factor kB
OPG: Osteoprotegerin
FGF23: Fibroblast growth factor 23
N: Nondominant
D: Dominant
CRT: Chair rising test
TUG: Timed up and go test.
3MWT: 3 min walking test
n.a.: Not applicable
SAb: Shoulder abduction
EF: Elbow flexion
EE: Elbow extension
HF: Hip flexion
KE: Knee extension
